# Formative Research Using Settings and Motives to Explore Child Faeces Disposal and Management in Rural Solomon Islands

**DOI:** 10.3390/ijerph19169815

**Published:** 2022-08-09

**Authors:** Adam Biran, Rosie Sanderson, Diana Gonzalez, Hugo Bugoro, Mohammad Kadir, David Gegeo, Jamesford Keboy, Clement Lifoia, Sheilla Funubo, Hellenda Honimae, Lanique Naolina Pitasua, Joanna Tatalu, Patishadel Jonah, Regina Souter

**Affiliations:** 1 Department of Disease Control, London School of Hygiene and Tropical Medicine, London WC1E 7HT, UK; 2 International Water Centre, Griffith University, Nathan 4111, Australia; 3 Department of Epidemiology and Research, Solomon Islands National University, Honiara P.O. Box R113, Solomon Islands; 4 Griffith Business School, Griffith University, Nathan 4111, Australia

**Keywords:** behaviour, sanitation, hygiene, children, gender, motives

## Abstract

Unsafe child faeces management can lead to adverse health and wellbeing outcomes for children. In Solomon Islands, diarrhoeal disease is a leading cause of under-5 mortality, though there is limited research into CFM practices and promotion of safe behaviours. The formative research applied a Behaviour-Centred Design framework to investigate the habits, motives and settings related to child faeces management in rural Solomon Islands villages. Data were collected through structured recall demonstrations by caregivers (*n =* 61), household infrastructure observations (*n =* 57), semi-structured interviews with caregivers (*n =* 121) and community leaders (*n =* 30), focus group discussions (*n* = 26), and three participatory activities with caregivers. The findings identified a range of CFM-related behaviours, some of which would be considered safe and some, such as outside defecation and disposal to a waterway, as unsafe. Convenience is important in shaping CFM practice and may help health benefits to be achieved without women bearing the cost of an increased work burden. Nurture and disgust may provide the basis for behaviour change communication in SI as they have elsewhere. Critically, the participation in and promotion of safe CFM by fathers in households should be promoted, and motivating such behaviours might be achieved through focus on nurture as a motive.

## 1. Introduction

Child faeces management (CFM) refers to a sequence of actions from the point of defecation to final disposal of faeces. Actions might include, for example, selecting a defecation place, moving the faeces to a disposal place using a tool or utensil selected for that purpose and cleaning the child. This is not an exhaustive list of actions. The sequence may be longer and more complicated or may be as short as defecating at a chosen location and walking away. The actions performed and the way(s) in which these are achieved can reduce or increase the probability of contact with faeces and the potential for faecal-oral disease transmission. For this reason, CFM is of public health concern. The faeces of young children are often believed harmless or less harmful than adult faeces, and this misconception has been associated with an increased risk of childhood diarrhoea in urban settlements in Papua New Guinea and also in rural settings globally [1,2,3]. Children’s faeces can contain higher concentrations of pathogens than adult faeces [4]. The perception of child faeces as harmless, along with barriers to accessing latrines, nappies, diapers, potties, and the resources to clean them, can lead to open defecation by children in the household environment, contaminating places where infants and children eat and play [2,5]. Exploratory behaviours of young children in a contaminated environment expose them to intestinal pathogens, as they frequently put objects, soil, and fingers into their mouths [6,7]. Safe CFM is intended to reduce the transmission of faecal pathogens, and while it is recognized that to do so requires consideration of a range of risk exposures [8,9] end disposal can be a key point of transmission. The World Health Organization (WHO) recognises disposal in a latrine or toilet as the only safe disposal method in the absence of formal solid waste management systems [10].

CFM in Solomon Islands (SI) has not been extensively explored in the academic or practitioner literature. Diarrhoeal disease is one of the leading causes of childhood mortality in SI, accounting for an estimated 12% of under-five mortality [11]. Rural household sanitation coverage is low (20%) and open defecation is common in rural areas [12]. The SI Rural Sanitation Policy uses Community Led Total Sanitation (CLTS) to promote sanitation uptake [13]. This participatory approach has been described in detail elsewhere [14,15]. It uses community visits by an external facilitator to raise awareness of the need for sanitation (‘triggering’) and, post-triggering, to support communities working towards declaration of ‘open defecation-free’ status (known in SI as ‘No Open Defecation’, NOD).

Experience elsewhere suggests that improvements to household sanitation have minimal effect on CFM practice, e.g., [16] and it has been argued that specific efforts are required to actively integrate promotion of safe CFM within sanitation programs [17]. Miller-Petrie, Voigt [18] found safer CFM to be associated with longer periods of latrine ownership among households in Cambodia, and propose that this reflects the development of safer routines over time. However, latrine uptake driven by CLTS may present an opportunity to improve CFM which could be realised through behaviour change communication. The current CLTS approach in SI lacks any element specifically targeting CFM.

Sanitation, hygiene and water (WASH) interventions frequently show strong, gender-based differences in the costs and benefits to recipients [19]. The case has been made that addressing gender-based inequalities and empowering women is central to improving child health [20]. Rural society in SI is known to have a prominent, gender-based division of labour and power and women are known to be routinely excluded from WASH resource management or decisions [21]. WASH interventions in SI should therefore take steps to address this where possible and to avoid compounding the situation.

In this paper we report a formative research study to inform the design of an intervention to promote safer and more gender-equitable CFM in SI. Education and awareness raising have not been found to be especially effective in changing WASH behaviours elsewhere, including CFM [22]. We therefore investigated other possible levers for change.

Our research was guided by the theoretical framework of Behaviour Centred Design, described in detail elsewhere [23,24]. Central elements of this framework are recognition of the importance of habit and motives in driving behaviour and of the influence of the behavioural ‘setting’, an interacting set of social and physical factors that can increase the probability of some behaviours and decrease that of others. Motives identified through formative research have previously been put to effect in hygiene and sanitation interventions, e.g., [25,26]. The concept of the behaviour setting was used in the design of a food hygiene intervention in Bangladesh [27] and, more recently, as a theoretical lens through which to examine domestic hygiene and water use in Nigeria [28]. Our objectives were to explore current CFM practice in rural Solomon Islands using the theoretical lens of behaviour ‘settings’, and to identify motives that might be used to encourage (i) use of latrines for disposal of child faeces and (ii) fathers’ involvement in CFM.

## 2. Materials and Methods

### 2.1. Study Design

We conducted a mixed methods qualitative study to explore behaviours associated with the disposal of child faeces.

### 2.2. Study Site

The study was carried out during 2020–2021 in rural areas of SI, a nation comprising nine provinces excluding the capital city, Honiara. SI has a total population of approximately 680,806 as of 2019 of which almost 75% are considered rural by the Solomon Islands National Statistics Office [29]. The country is divided into provinces as the largest, sub-national, administrative units. A province typically constitutes an entire island (or multiple islands) and may be culturally distinct from other provinces.

The study was conducted in five rural villages across two provinces, Guadalcanal and Isabel, where Community-Led Total Sanitation (CLTS) had been implemented in some communities, by a range of implementers. In both provinces, children aged 0–4 years comprised and estimated 12% of the population [30].

### 2.3. Sampling and Recruitment

We used the Solomon Islands Ministry of Health and Medical Service’s Rural Water, Sanitation and Hygiene (RWASH) Unit’s database of communities that have participated in CLTS to guide the selection of provinces and villages. The database listed communities in six provinces. Guadalcanal and Isabel were selected purposively for ease of access, socio-cultural diversity and because we were able to engage with the Civil Society Organisations (CSOs) responsible for facilitating CLTS in those provinces.

In each province, we shortlisted villages from the RWASH database using the following criteria, (1) declared as having no open defecation (NOD) status or have made progress towards NOD; (2) listed as ‘rural’ by RWASH; (3) comprising more than 15 households of which more than five households had children under five years old.

We were informed by the CLTS implementers in Guadalcanal and Isabel that three additional villages met these criteria but had not yet been added to the database. These were added to the shortlist to give us a total of 18 villages. We selected the final five villages purposively, based on accessibility, population size and personal contacts to facilitate an introduction into the village (Table 1).

In each village, all households with children under five years old were identified by the village chief or a village research assistant (VRA) engaged for the project. These households were invited to participate in the structured-recall demonstration, sanitation and hygiene infrastructure spot-checks and focus group discussions. From these initial activities, in each village researchers purposively sampled two mothers who reported disposing of their children’s faeces in a latrine and two who did not, and two fathers who reported participating in CFM and two who did not. Selection was based on availability and respondents’ apparent confidence and willingness to contribute to discussion. These individuals were invited to participate in semi-structured interviews and associated activities to explore motives, knowledge, beliefs and practices, and village mapping. Additional respondents were recruited on an ad-hoc basis. Individual respondents commonly participated in more than one data collection activity. Multiple respondents from the same household were permitted to participate in the study. Additional, key informants were selected from the village leadership, including the Chief and committee members.

### 2.4. Data Collection

We collected data in Isabel Province in February and March 2020 and in Guadalcanal Province in February 2021. Four researchers (two female) spent four or five days in each village. Data were collected through structured recall demonstrations, spot-check observations, semi-structured interviews, key informant interviews and small group discussions with three to six participants). Individual data collection activities were normally conducted outside the respondent’s home, while group activities were within village public places such as meeting houses. The duration of different data collection activities varied, however, typically an interview or demonstration with a single respondent took approximately 1 h while group discussions lasted around 90 min. Participatory activities were used to help stimulate or guide the discussion or conversation. Summary descriptions of data collection tools are presented in Table 2.

### 2.5. Data Recording and Analysis

Data were collected as notes taken during interviews and FGDs, comprising summaries of responses to critical questions and illustrative verbatim quotes. Summary notes were transcribed into analysis matrices to allow review of data within, and comparison across, cases, following Richie and Spencer’s Framework Analysis approach [31]. These matrices were used as the basis for an inductive thematic analysis in MS Excel based on themes derived from the BCD framework. Thematic analysis was led by three of the authors (AB, DG and RPS). Their analysis was reviewed, checked and commented on by all other authors and a consensus on the interpretation and conclusions was reached through. The authors did not use any qualitative data analysis software.

We summarized and presented our results using a framework based on the BCD model considering *elements of the behaviour setting (stage, routines, infrastructure, props, roles, competencies, and norms) as* well as considering the rational planning and motivated brain systems. Following Aunger and Curtis [24] and Curtis et al. [29], summary definitions of these elements are presented in Table 3.

## 3. Results

### 3.1. Respondent Characteristics

The study included 161 individual respondents, some of whom participated in multiple data collection activities. Respondents included mothers (72), fathers (48), grandmothers (19), and community leaders (22).

In this case, 61 parents (mothers and fathers) from 57 households participated in the structured CFM recall demonstration. Household sanitation and handwashing access was assessed through spot-check observations, as outlined in Table 2. The characteristics of these respondents are shown in Table 4.

### 3.2. Stage

Children’s defecation places varied with the child’s age. In our sample, defecation indoors and the use of nappies was confined to children below two years and for children aged two to three years, defecation was most commonly on the ground outside the house.

Demonstrated CFM practices took place close to the house, usually within an area comprising the compound or yard but sometimes incorporating common areas such as the bush, sea, beach or a water course in households that were close to these. Designated open defecation (OD) areas were mentioned in one village as a disposal place and in others as a place for adults to defecate, however there did not appear to be a commonly designated disposal place for children’s faeces in most villages in the study. Respondents mentioned the importance of proximity and convenience in influencing choice of disposal place.

*‘[disposal in the river is…] an easy practice.’*

Mother, Isabel province

*‘It is convenient and easy to throw them in the bush.’*

Father, Isabel province

*“Before we came down to this place, we stayed on the hill, so we practiced dry pit [sanitation]. But when we came down near the sea, we settled beside this water, and we use the water to defecate.”*

Mother, Isabel province

### 3.3. Routines

We distinguished seven actions in CFM: defecation, faeces transport, faeces disposal, cleaning utensils, storing soiled utensils, cleaning child, and handwashing with soap. These are defined below.

- *Defecation:* The child defecates. The faeces enter the environment. Sometimes at a location chosen by the caregiver, sometimes chosen by the child, with or without the knowledge of the caregiver.
- *Faeces transport:* The faeces are moved from the place of defecation.
- *Faeces disposal:* The faeces are placed somewhere to reduce the probability of human contact, sight or smell.
- *Cleaning of child:* Anal cleansing of the child who has defecated, sometimes accompanied by cleaning legs and buttocks. Always with water. Sometimes with soap.
- *Cleaning of utensils:* Implements or cloths used to move faeces are rinsed or washed.
- *Storage of soiled utensils:* Implements (particularly clothes or cloth) are temporarily stored for later washing, such as storage overnight in a bucket.
- *Handwashing:* Hands of caregiver and/or child are rinsed or washed. Always with water. Sometimes with soap.

Respondents varied in which of these actions they performed and in what way (see Table 5). In this case, 30 respondents (49%) demonstrated use of a latrine as the final disposal point. In 13 of these households the child used the latrine as the place of defecation. Disposal or defecation in the bush, sea, or river was reported by 19 participants (31%). Sometimes this was the site at which the child defecated. More common was for the faeces to be transported there for disposal.

Respondents also varied in the sequencing of actions demonstrated. Most respondents demonstrated a sequence of actions in which faeces were first transported and disposed of, then the child cleaned. A minority of demonstrations reversed this, beginning with cleaning the child. For sequences that incorporated cleaning utensils and/or handwashing, these occurred at or near to the end of the sequence. Table 6 shows the frequency with which different activities occurred at each step of the sequence.

### 3.4. Infrastructure

Among households with latrines, dry pits and pour-flush variants were seen with similar frequency. (Table 4). The dry pits that we observed had wooden floors and some were fitted with drop-hole covers. Some parents disliked dry pits were because of the smell and flies.

Latrines were commonly constructed around 20 m or more from houses, sometimes up hill. Respondents noted that this can present a barrier to the use of latrines for child faeces disposal.

*“We have no proper place to dispose of the poo and the distance from the house to the shared toilet is far”*

—Mother, Guadalcanal province.

### 3.5. Props

A variety of utensils or scrap materials were used for the transport of faeces including spades, coconut husks and cardboard toilet paper, cloth, water, and soap were all sometimes used for cleaning children. Some caregivers reported that the convenience influenced whether they were used or not. For example, if the spade were not in sight, caregivers may not go searching for it but would instead use a more conveniently placed alternative. Respondents also noted that they disliked the additional effort required to dispose of materials used to carry faeces to the latrine, such as cardboard, leaves, or latrine paper and some were concerned that these might cause latrine blockages or filling of latrines.

The use of disposable nappies was rare, (2 of 61 demonstrations) and no respondents reported use of a potty. Cloths were used to clean children and transport faeces and were rarely worn by children as nappies. Some respondents also mentioned the use of a bucket to store and transport soiled clothes to the laundry site.

### 3.6. Roles

Roles associated with CFM included the child who defecated, the caregiver who responded by cleaning the child and disposing of the faeces. Sometimes another person, usually a sibling of the young child, made the caregiver aware that defecation had occurred. Sometimes, a second caregiver assisted with some steps in the sequence, such as a mother washing a soiled cloth that had been placed in a bucket by the father, or a father moving the faeces while the mother cleaned the child.

### 3.7. Competencies

Children’s competencies increased with age. Defecation on the floor of the house was not reported in recall demonstrations by caregivers of children over 23 months old, nor was the use of diapers reported for this age group. Children generally began to use the latrine with assistance from their caregivers at about three years of age; only two children younger than three were reported to use the latrine for defecation.

### 3.8. Social Environment: Norms Relating to Disposal Practices

When asked whether the opinion of other people mattered with respect to their CFM practice, mothers reported they were primarily concerned about the views of their husband because the husband oversaw the household. The opinions of parents and in-laws (particularly mothers-in-law) and neighbours were also important. Fathers reported that the opinions of their mothers, mothers-in-law and wives might matter. Some also mentioned the opinion of their friends.

There was a widely recognised injunctive norm against leaving faeces lying around. Leaving faeces lying was reported to carry the risk of social sanctions such as being thought of poorly or gossiped about by neighbours. There was variation in the reported injunctive norms of disposal place. When caregivers were asked what they thought *should* be carried out with children’s faeces, responses included: disposing of faeces in latrines, burying them and throwing them in rivers. Some respondents recognised injunctive and behavioural norms of disposing of child faeces in a latrine, and one respondent mentioned the possibility of formal sanctions for not doing so.

*“Most people dispose of faeces in the latrine, especially those who have toilets. For those like us who have problems with our toilet we do it in the mangrove, but we hide it from the community elders. If they know that we practice open defecation again, we will be fined for the practice.”*

Mother, Isabel province.

Female respondents suggested that a hypothetical woman who used a latrine for faeces disposal would be ‘clean, caring, loving and educated, responsible, trustworthy, hardworking, respected and neat’ and that their home would be clean, and their children cared for. A hypothetical woman who disposed of faeces elsewhere was described as lazy, incompetent and irresponsible, with a dirty domestic environment and an uncooperative family.

*“If the mother does not clean her child’s poo whenever the child defecates around the house and her aunties come over to the house and see the poo lying around, they will shout at the mother and call her lazy and tell her to clean it up.”*

Mother, Isabel province

### 3.9. Social Environment: Gender-Related Norms

Respondents of both genders described gender norms relating to domestic tasks including CFM. A small number of fathers reported that CFM was only the mother’s responsibility—most men reported it was a shared responsibility to feed and care for their children, including cleaning them and disposing of faeces. However, some women reported that men would never do CFM activities and might become angry if their wife asked them to do so. Men and women reported an increased involvement of fathers in the present when compared with previous times. Men reported that prior to the arrival of the church, men would not have handled children’s faeces due to the belief that this could bring bad luck in fishing or hunting, but that now these beliefs no longer held and that men may play a role in CFM.

*“I never heard much about (fathers’ involvement) from my great grandparents. Because … men during those days would not tolerate (helping with CFM) because they believed that those practices … would cause them to be impure to their gods. So, men were not allowed to practice (CFM), only women. So yes, before men did not usually clean children’s poop compared to today where men participate in cleaning children’s poop, disposing of it and doing other roles that were considered for women before.”*

Father, Isabel province.

Nevertheless, it was reported that a man’s relatives might complain if they saw him cleaning up children’s faeces while his wife was present (and thus considered responsible). It was also thought that people might gossip about a woman being lazy if her husband were to clean up child’s faeces while she was present, as this was not regarded as an appropriate task for the head of the household.

*“If my wife asked me to clean the child when she was doing nothing, I would be offended and mad, and tell her that it’s not my role, its women’s role. But if the mother busy doing anything, or sick and no one is around then I can help to do that but only for picking-up feces and cleaning up child’s bottom—never laundry.”*

Father, Guadalcanal province.

Men and women expressed the opinion that men should be more involved in CFM, but that it was generally not socially acceptable for a man to do this if his wife were able to perform the task, and that male involvement in CFM would be associated with possible loss of status for men. However, male respondents agreed that their participation in CFM would be beneficial for their child and expressed a willingness to practice CFM.

It was noted by some, that male participation in CFM could be praiseworthy. A hypothetical man who participated in CFM was regarded positively by both men and women. This man was described as ‘good, clean, cooperative, willing, helpful, supportive and caring’, while a man who did not participate in CFM was described in opposite terms as ‘unsupportive, lazy, uncooperative’.

Men associated male participation in CFM with a hypothetical household that was cleaner, wealthier and better organised, because the sharing of labour helped facilitate these achievements. In contrast, female respondents suggested that lack of male participation in household tasks such as CFM would be associated with increased cooperation among other family members to compensate for the lack of participation by the husband/father. These respondents also suggested that male involvement in CFM could be indicative of failure by women in these households to perform their domestic duties adequately. Thus, they suggested that in the households where men were active in CFM, children may not be cared for properly and the house may be untidy or unclean.

### 3.10. Rational Planning: Knowledge about Child Faeces and Disease Transmission

There was awareness of germs and of a possible health risk from contact with faeces and of the possible role of flies in spreading faecal contamination. Faeces were also recognised as potential sources of intestinal worms and were thought to cause diarrhoea as well as non-intestinal health conditions including itchy skin, eye infections and upper respiratory tract infections.

*“… if the river isn’t flowing, germs from the poo will stay in the water and spoil the water this will cause skin itchiness and disease if people touch or swim in the water.”*

Mother, Isabel province.

However, there was a belief that the faeces of older children and adults were more harmful than those of young children and babies.

*“I was told by my mother that infant’s faeces are not harmful, so it is okay to dispose/wash them of in the river”*

Mother, Guadalcanal province.

### 3.11. Motives

Through the ‘Motive Mapping’ exercise, female respondents identified **nurture** and **affiliation** as the motives they believed to be most strongly and plausibly underlying the use of a latrine for faeces disposal and indicated that love (for or from their husband or partner) was the least believable. One mother, when asked about why a parent would practice safe CFM, responded:

*“Because mothers always think about our child’s wellbeing—always our children come first”*

Mother, Guadalcanal province.

Another respondent told the researchers:

*“I will feel ashamed because I am not doing the same thing as the rest of the community. The community will talk about me, and they will not be happy with me”*

Mothers Group respondents, Isabel province

When we explored fathers’ motives for becoming involved in CFM, the **nurture** motive was the predominant explanation, sometimes expressed as a desire to protect their children and keep them safe.

“(Some think that) *men should not be involved in any role that deals with child’s faeces. Others might talk* (gossip) *about a man who does get involved, however, as a father, he probably cares for his child and would just do it anyway”*.

Father, Guadalcanal province.

When responding to a question of which types of man would become involved in CFM for their children, responses included:

*“A type of man that never cares about the culture/custom first, but rather cares and loves his child for healthy living”*

Father, Guadalcanal province.

*“(The) type of man that are willing to do anything for the wellbeing of the family. They are always willing to help their wife and keep their family healthy”.*

Father, Isabel province.

Interview respondents—both mothers and fathers—reported a disgust-based response regarding children’s faeces in the domestic environment; this was manifest as a desire to avoid the sight and smell of faeces. However, there was a less strong disgust response associated with the faeces of one’s own child in comparison to those of other children, and children’s faeces were considered by many respondents as less disgusting than those of adults, and to be less disgusting the younger the child. Some fathers wanted to avoid embarrassment (loss of status) when visitors came, and thus made sure faeces were not present in the yard.

Disposal of faeces in a latrine was appreciated because it helped prevent contamination of the domestic environment by flies and because it removed the sight and smell of faeces, all of which were considered disgusting. Similar benefits were associated with disposal of faeces in bodies of water.

*“…when poo is lying around the house, it looks disgusting so the best thing to do is to bury and hide it”*.

Mother, Isabel province.

*“If the poo cannot be removed immediately the mother might forget to clean it. This will cause bad smell for the family”*

Father, Guadalcanal province.

Similar benefits were associated with disposal of faeces in bodies of water.

*“…if the faeces are disposed of in the water the faeces will wash down the stream but when it’s disposed of in the bush the faeces will stay there and when people go there, they will step on it.”*

Father, Isabel province.

However, some respondents noted the risk of coming back into contact with faeces that were disposed of in this way.

*“If we throw it in the sea sometimes a wave can take it back to the seashore and children or we can step on it again”*.

Mother, Isabel province.

## 4. Discussion

As in previous findings from other countries, e.g., [16,18] our data suggest that child faeces management in the Solomon Islands remains sub-optimal from a public health perspective, with unsafe disposal remaining widespread even as efforts to improve rural sanitation coverage increase.

The sequence of CFM actions we observed during CFM structured-recall demonstrations (transporting; disposing of faeces; and cleaning child, utensils and hands) were similar to those described by Miller-Petrie, Voigt [18] in Cambodia. Considering CFM behaviour in this way can help highlight potential risk or protective practices within the sequence, such as washing or not washing hands or utensils. In contrast to the order of the sequence of actions seen in Cambodia, respondents in our study commonly demonstrated transporting and disposing of faeces *prior* to cleaning the child. Further work might explore whether public health and behaviour change implications are associated with this difference in sequencing.

Each participant in the CFM demonstrations was asked to recall and demonstrate their practice on a single occasion (the ‘last time’ they had accomplished this). We do not know the extent to which demonstrations were based on recall of this specific occasion rather than a more generalised recall of ‘typical’ practice. Either way, the technique was not well-suited to recording intra-individual variation in practice. It seems likely that the timing and location of child defecation and the availability and location of props and infrastructure relative to defecation place may vary between defecation events as well as between households/caregivers. The consistency and quantity of faeces, the presence or absence of guests or other family members and the extent and nature of competing caregiver priorities may also vary. Any of these might serve as cues, facilitators or constraints leading to variation in CFM behaviours.

Changes in CFM practice may also occur as the child’s age increases. Our small and non-random sample precludes much by way of subgroup analysis. Nevertheless, our data suggest age-related variation in defecation location, use of diapers and frequency of latrine use.

Considering the variation we observed, the CFM action sequence in SI, may be flexible rather than constituting a ‘routine’ in the sense used by Curtis et al. [28] of a repeated, semi-automated behaviour sequence in which the actions, timing and location remain constant. The scope for variation in the CFM behaviours we saw might mitigate against habit formation and automaticity. Alternatively, a more fine-grained approach to data collection and analysis might reveal the CFM sequence to be composed of multiple, sub-routines which remain relatively constant, but which are selected or omitted in response to combinations of cues, facilitators and constraints in the behaviour setting. Future work could explore this and the associated implications for behaviour change.

Exploring the CFM setting helped highlight the influence of props and infrastructure and their location relative to actors. This was explained by some respondents as the importance of ‘convenience’. It influenced the choice of disposal place. The river the sea and the bush were perceived as convenient by respondents living close to these and even respondents with prior experience of latrine ownership chose to use the sea when it was close by. A similar effect was noted by Marjorin et al. [32] who reported that Indian households with latrines closer to the house were more likely to use these for CFM. Convenience may also have influenced the choice of transportation materials.

Altering the setting by making latrines and/or transport materials more convenient might be one route to influence safer practice [33,34]. The settings in which CFM was demonstrated were not designed primarily to facilitate safe CFM. Other actions take place within the same physical space, sometimes sharing the same props and infrastructure. These include elements of socialising, food preparation, storage of items, laundry and other domestic tasks. This sharing of space might increase the health risks posed by unsafe CFM. At the same time, features of the setting, such as the design and location of latrines and water sources (particularly where located at a distance from the house and/or water access location) may be intended to be optimal for purposes other than safe CFM and may serve to inhibit rather than facilitate safe CFM practice.

Interventions which make desired practice more convenient may additionally reduce, rather than increase, the burden of domestic labour placed on women and therefore contribute to gender equity in domestic tasks. This principle is readily recognised with respect to water supply [35,36,37] and is equally applicable to CFM interventions.

Previous formative research studies have found WASH practices to be associated with the motives of disgust and nurture, e.g., [38,39]. In SI we found evidence for nurture as a driver for mothers and fathers to engage in CFM. For fathers, in some circumstances, this could override gender-based norms, allowing fathers to engage with CFM even when their wives were otherwise present. Furthermore, nurture has been used as the basis for an intervention in India which reported some success in increasing safe child faeces disposal by caregivers [40]. We also found some evidence of disgust associated with faeces and flies and desire to avoid these cues of contamination, though this was usually with respect to others’ faeces, and not directly towards the faeces of their own child when performing CFM actions.

Some of our findings are based on one-off demonstrations of recalled, self-reported CFM practice. These data are susceptible to recall and desirability/courtesy bias and may fail to capture individual variation in practice and the different environmental and social factors associated with this variation. Future studies should collect repeated, observations of actual disposal practices.

## 5. Conclusions

Our study found that a range of CFM behaviours are practiced by caregivers in rural Solomon Islands, some of which could be considered safe, and some that are unsafe with respect to potential pathogen transmission. Defecation by children younger than 5 years old outside the house was found to be common, and almost half of our study respondents disposed of faeces to unsafe locations. There is some misunderstanding among parents in rural areas in Solomon Islands about the health risks posed by child faeces. However, this is not likely to be key in driving or changing practice. Convenience is important in shaping CFM practice and may help health benefits to be achieved without women bearing the cost of an increased work burden. Use of a latrine for child defecation simplifies the CFM sequence and likely reduces the risk of faecal contact, bringing associated health benefits. Improving the quality and convenience of latrines to support and facilitate their use by younger children would be a useful element of a CFM intervention in SI and might be combined with communication to encourage early toilet training of children.

Nurture and disgust may provide the basis for behaviour change communication in SI as they have elsewhere. There is also a need to influence attitudes towards male involvement in CFM, both among fathers and among their relatives. The nurture motive may offer one route towards this. The nurture motive is already seen as legitimising fathers’ involvement in CFM and could be used in communication as a way of adding value to the desired practice. Our findings echo those of previous studies, e.g., [16,18] that the disposal of child faeces is not a single behaviour but a behavioural sequence. Future research might usefully explore the extent and nature of variation within this sequence and whether its constituent behaviours form stable sub-routines associated with characteristics of the behaviour setting. Our findings are consistent with those of previous studies, e.g., [16,40] in highlighting the likely importance of appropriate sanitation hardware and theory-based behaviour change communication in efforts to improve CFM practice for public health benefit.

## Figures and Tables

**Table 1:** Characteristics of the five villages within the formative study.

	Village 1	Village 2	Village 3	Village 4	Village 5
Village population ^1^	70	248	409	113	150
Location	Isabel province, inland	Isabel province, coastal	Isabel province, inland	Guadalcanal province, inland	Guadalcanal province, coastal
CLTS ^2^ status	NOD ^3^ (2019)	NOD (2017)	Triggered (2017)	NOD (2016)	NOD (2021)

^1^ Estimated by CLTS facilitating organisation. ^2^ Community-Led Total Sanitation. ^3^ No Open Defecation (open-defecation free).

**Table 2:** Data collection tools.

Tool	Sample Size	Description
Structured-recall demonstration	61 respondents from 57 households	Caregivers of children under five years were asked to recall the last time the youngest child defecated and to demonstrate the sequence of steps they took in response. We asked respondents (42 mothers and 19 fathers) to demonstrate the sequence of CFM beginning with defecation by the child and ending at the point that the respondent went on to an activity not related to child faeces. Observers recorded the sequence of events (routine) and observable features of the setting (infrastructure, props), and used the demonstration as a prompt to question the caregiver about the behaviours demonstrated and about possible social influences on these.
Spot check observation	57 households	Characteristics of household sanitation, hygiene and water infrastructure were recorded using a predefined checklist.
Semi-structured interviews (SSI)	121 respondents	Interviews with individual caregiver respondents, following a set of open-ended questions with the order, prompting, probing and additional lines of questioning applied at the discretion of the interviewer. Interviews took place with male and female respondents (35 fathers, 78 mothers, and eight grandmothers) and were used to explore the same topics as described for focus group discussions.
Key informant interviews	30 respondents	SSIs with village chiefs (4), leaders and representatives of the WASH or CLTS committee (8), and household members with experience of CLTS (18)
Small group discussion	26 groups	Discussions with small, single-gender groups, 14 with mothers, eight with fathers and four with grandmothers, used to explore a variety of topics including: gender roles, knowledge and attitudes relating to child faeces management, social norms relating to child faeces management.
Motive mapping	52 respondents in 9 groups, 7 individuals	A projective technique using picture cards illustrating different child faeces disposal practices and motives, allowing respondents to ascribe motives to practices and to discuss the plausibility of associating specific motives with safe or unsafe practices. This was done with respondents in small groups and individually, as separate activities to the initial FGDs, but with similar respondents.
Doer/Non-doer attributes	9 respondents	A projective technique whereby respondents attribute individual and household characteristics to hypothetical ‘doers’ (men who participate in child faeces disposal/women who use a latrine for child faeces disposal) and ‘non-doers’ (men who do not participate in child faeces disposal/women who do not use a latrine for child faeces disposal). This was done with respondents in small groups and individually, as separate activities to the initial FGDs, but with similar respondents.
Village mapping	5 groups	A participatory tool whereby respondents, in a single-gender group separately to the initial FGDs, work together to draw a map of their village, marking features of potential interest to the project, such as water sources, waste disposal sites and defecation sites.

**Table 3:** Summary definitions of the BCD elements.

BCD Element	Summary Definition
Stage	The immediate location (in and around the home) where demonstrated CFM activities were observed
Routines	Sequences of behaviour performed regularly
Roles	The behaviour of individuals contributing to the overall CFM sequence
Competencies	Physical and cognitive abilities that allow individuals to perform CFM actions
Infrastructure	Technologies or manmade features of the stage used to complete the CFM actions.
Props	Objects manipulated and used to complete CFM actions.
Social Environment: Norms	Implicit social rules
Rational Planning	A brain system that uses information to compare different possible outcomes, inform choices and influence behaviour in pursuit of longer-term goals
Motivated Brain	A brain system that directs behaviour towards short to medium-term goals that are driven by one or more of 15 human motives

**Table 4:** Respondent and household characteristics for structured recall demonstration.

Characteristic		
Age (years)	Mean	29.6
	Median	30.0
	Range	20–45
Gender	Female	42 (69%)
	Male	19 (31%)
Number of children in household	Mean	2.3
	Median	2.0
	Range	1–6
Age of youngest child (months)	Mean	20.2
	Median	23.0
	Range	1–60
Household sanitation infrastructure—type	None	14 (25%)
	Dry pit latrine	24 (42%)
	Pour-flush latrine	19 (33%)
Household sanitation infrastructure—functionality	Latrine is functional, stable, clean, and not dark	4 (7%)
Household handwashing infrastructure	Water and soap	14 (23%)
	Water only	29 (48%)
	None	14 (23%)

**Table 5:** CFM actions recalled and demonstrated.

Action	Description	Frequency (*n* = 61)
Defecation (location)	Outside house on ground	28 (46%)
	Toilet/latrine	13 (21%)
	Floor inside house	10 (16%)
	Diaper/nappy	5 (8%)
	Outside in river, stream or on beach (near waterway)	3 (5%)
	In clothes	1 (2%)
	In designated open-defecation area	1 (2%)
Faeces transport (utensils/materials used)	Spade	16 (26%)
	Cardboard, paper, cloth, toilet paper	7 (11%)
	Reusable nappy	7 (11%)
	Leaves, coconut shell, grass	6 (10%)
	Clothes	3 (5%)
	Disposable diaper	2 (3%)
	Waste plastic packaging	1 (2%)
	None (faeces not transported)	19 (31%)
Faeces disposal (location)	Latrine (transported to latrine)	17 (28%)
	Latrine (defecation in latrine)	13 (21%)
	Thrown in sea, beach, river, stream or drain	8 (13%)
	Thrown in bush or left at OD site	7 (11%)
	Buried	6 (10%)
	Washed from cloth/nappy	4 (7%)
	Defecated and left in sea, beach, river, stream or drain	4 (7%)
	Garbage	2 (3%)
Cleaning child	Child’s bottom washed	58 (95%)
Cleaning utensils	Material or utensil washed	16 (26%)
Handwashing	Respondent’s hands washed	20 (33%)
	Child’s hands washed	18 (30%)

**Table 6:** Sequence of CFM actions (following defecation outside of the latrine) as demonstrated by respondents.

	Transport	Disposal	Cleaning Child	Cleaning Utensils	Hand Washing
First action taken	30 (63%)	11 (2%)	17 (35%)	0	0
Second action taken	13 (27%)	30 (63%)	1 (2%)	0	4 (8%)
Third action taken	0	12 (25%)	30 (63%)	0	0
Fourth action taken	0	0	0	16 (33%)	9 (18%)
Fifth action taken	0	0	0	0	1 (2%)
Action not performed	5 (10%)	5 (10%)	0	32 (67%)	35 (71%)

## Data Availability

The data presented in this study are available on reasonable request from the corresponding author. The data are not publicly available due to privacy requirements under our ethics approvals.

## References

[1] Bukenya G., Kaser R., Nwokolo N. (1990). The relationship of mothers’ perception of babies’ faeces and other factors to childhood diarrhoea in an urban settlement of Papua New Guinea. Ann. Trop. Paediatr..

[2] Gil A., Lanata C., Kleinau E., Penny M. (2004). Children’s Feces Disposal Practices in Developing Countries and Interventions to Prevent Diarrheal Diseases: A Literature Review.

[3] Curtis V., Kanki B., Mertens T., Traore E., Diallo I., Tall F., Cousens S. (1995). Potties, pits and pipes: Explaining hygiene behaviour in Burkina Faso. Soc. Sci. Med..

[4] Brown J., Cairncross S., Ensink J.H.J. (2013). Water, sanitation, hygiene and enteric infections in children. Arch. Dis. Child..

[5] Islam M., Ercumen A., Ashraf S., Rahman M., Shoab A.K., Luby S.P., Unicomb L. (2018). Unsafe disposal of feces of children <3 years among households with latrine access in rural Bangladesh: Association with household characteristics, fly presence and child diarrhea. PLoS ONE.

[6] Curtis V., Cairncross S., Yonli R. (2000). Review: Domestic hygiene and diarrhoea—Pinpointing the problem. Trop. Med. Int. Health.

[7] Majorin F., Torondel B., Chan G.K.S., Clasen T. (2019). Interventions to improve disposal of child faeces for preventing diarrhoea and soil-transmitted helminth infection. Cochrane Database Syst. Rev..

[8] Bauza V., Majorin F., Routray P., Sclar G.D., Caruso B.A., Clasen T. (2020). Child feces management practices and fecal contamination: A cross-sectional study in rural Odisha, India. Sci. Total Environ..

[9] Majorin F., Torondel B., Routray P., Rout M., Clasen T. (2017). Identifying Potential Sources of Exposure Along the Child Feces Management Pathway: A Cross-Sectional Study Among Urban Slums in Odisha, India. Am. J. Trop. Med. Hyg..

[10] World Health Organization (WHO) (2018). Guidelines on Sanitation and Health.

[11] Liu L., Oza S., Hogan D., Chu Y., Perin J., Zhu J., Black R.E. (2016). Global, regional, and national causes of under-5 mortality Fin 2000–15: An updated systematic analysis with implications for the Sustainable Development Goals. Lancet.

[12] World Health Organization (WHO) (2020). Health at a Glance: Asia/Pacific 2020 Measuring Progress towards Universal Health Coverage: Measuring Progress towards Universal Health Coverage.

[13] Government of Solomon Islands (2017). Solomon Islands Detailed National Sustainable Sanitation Plan.

[14] Kar K., Chambers R. (2008). Handbook on Community-Led Total Sanitation.

[15] Venkataramanan V., Crocker J., Karon A., Bartram J. (2018). Community-led total sanitation: A mixed-methods systematic review of evidence and its quality. Environ. Health Perspect..

[16] Freeman M.C., Majorin F., Boisson S., Routray P., Torondel B., Clasen T. (2016). The impact of a rural sanitation programme on safe disposal of child faeces: A cluster randomised trial in Odisha, India. Trans. R. Soc. Trop. Med. Hyg..

[17] Sahiledengle B. (2019). Prevalence and associated factors of safe and improved infant and young children stool disposal in Ethiopia: Evidence from demographic and health survey. BMC Public Health.

[18] Miller-Petrie M.K., Voigt L., McLennan L., Cairncross S., Jenkins M.W. (2016). Infant and young child feces management and enabling products for their hygienic collection, transport, and disposal in Cambodia. Am. J. Trop. Med. Hyg..

[19] Fisher J., Cavill S., Reed B. (2017). Mainstreaming gender in the WASH sector: Dilution or distillation?. Gend. Dev..

[20] Cavill S., Mott J., Tyndale-Biscoe P., Bond M., Huggett C., Wamera E. (2018). Engaging men and boys in sanitation and hygiene programmes. Frontiers of CLTS: Innovations and Insights 11.

[21] Alexander K.T., Baron R., UNICEF (2018). Because She Is Important. Actions for Gender Equity in Rural WASH: Solomon Islands.

[22] Morita T., Godfrey S., George C.M. (2016). Systematic review of evidence on the effectiveness of safe child faeces disposal interventions. Trop. Med. Int. Health.

[23] Aunger R., Curtis V. (2014). The evo–eco approasch to behaviour change. Applied Evolutionary Anthropology.

[24] Aunger R., Curtis V. (2016). Behaviour Centred Design: Towards an applied science of behaviour change. Health Psychol. Rev..

[25] Biran A., Schmidt W.-P., Varadharajan K.S., Rajaraman D., Kumar R., Greenland K., Curtis V. (2014). Effect of a behaviour-change intervention on handwashing with soap in India (SuperAmma): A cluster-randomised trial. Lancet Glob. Health.

[26] Greenland K., Chipungu J., Curtis V., Schmidt W.-P., Siwale Z., Mudenda M., Chilengi R. (2016). Multiple behaviour change intervention for diarrhoea control in Lusaka, Zambia: A cluster randomised trial. Lancet Glob. Health.

[27] Gautam O.P., Schmidt W.-P., Cairncross S., Cavill S., Curtis V. (2017). Trial of a novel intervention to improve multiple food hygiene behaviors in Nepal. Am. J. Trop. Med. Hyg..

[28] Curtis V., Dreibelbis R., Buxton H., Izang N., Adekunle D., Aunger R. (2019). Behaviour settings theory applied to domestic water use in Nigeria: A new conceptual tool for the study of routine behaviour. Soc. Sci. Med..

[29] Government of Solomon Islands (2015). Household Income and Expenditure Survey Report (HIES).

[30] Government of Solomon Islands (2009). Census of Population and Housing.

[31] Ritchie J., Spencer L., Huberman M., Miles M.B. (2002). Qualitative data analysis for applied policy research. The Qualitative Researcher’s Companion.

[32] Majorin F., Nagel C.L., Torondel B., Routray P., Rout M., Clasen T.F. (2019). Determinants of disposal of child faeces in latrines in urban slums of Odisha, India: A cross-sectional study. Trans. R. Soc. Trop. Med. Hyg..

[33] Neal D., Vujcic J., Burns R., Wood W., Devine J., Scudder P. (2016). Nudging and Habit Change for Open Defecation: New Tactics from Behavioral Science.

[34] Dickinson K.L., Patil S.R., Pattanayak S.K., Poulos C., Yang J.-H. (2015). Nature’s call: Impacts of sanitation choices in Orissa, India. Econ. Dev. Cult. Chang..

[35] Ferrant G., Pesando L.M., Nowacka K. (2014). Unpaid Care Work: The Missing Link in the Analysis of Gender Gaps in Labour Outcomes.

[36] Koolwal G., Van de Walle D. (2013). Access to water, women’s work, and child outcomes. Econ. Dev. Cult. Chang..

[37] Fisher J. (2006). For Her It’s the Big Issue: Putting Women at the Centre of Water Supply, Sanitation and Hygiene.

[38] Greenland K., Iradati E., Ati A., Maskoen Y.Y., Aunger R. (2013). The context and practice of handwashing among new mothers in Serang, Indonesia: A formative research study. BMC Public Health.

[39] White S., Thorseth A.H., Dreibelbis R., Curtis V. (2020). The determinants of handwashing behaviour in domestic settings: An integrative systematic review. Int. J. Hyg. Environ. Health.

[40] Caruso B.A., Sclar G.D., Routray P., Nagel C.L., Majorin F., Sola S., Koehne W.J., Clasen T. Effect of a Low-Cost Behaviour Change Intervention on Latrine Use and Safe Child Faeces Disposal in Rural Odisha, India: A Cluster-Randomized Controlled Trial. Prepr. THE LANCET.

